# Arginine promotes the activation of human lung fibroblasts independent of its metabolism

**DOI:** 10.1042/BCJ20253033

**Published:** 2025-06-17

**Authors:** Robert B Hamanaka, Kun Woo D Shin, M Volkan Atalay, Rengul Cetin-Atalay, Hardik Shah, Jennifer C Houpy Szafran, Parker S Woods, Angelo Y Meliton, Obada R Shamaa, Yufeng Tian, Takugo Cho, Gökhan M Mutlu

**Affiliations:** 1 Department of Medicine, Section of Pulmonary and Critical Care Medicine, The University of Chicago, Chicago, IL 60637, U.S.A.; 2 Department of Information Systems and Supply Chain Management, Loyola University Chicago, Chicago, IL 60611, U.S.A.; 3 Metabolomics Platform, Comprehensive Cancer Center, The University of Chicago, Chicago, IL 60637, U.S.A.

**Keywords:** arginine, fibroblast, metabolism, pulmonary fibrosis, transforming growth factor-β

## Abstract

Arginine is a conditionally essential amino acid with known roles in protein production, nitric oxide synthesis, biosynthesis of proline and polyamines, and regulation of intracellular signaling pathways. Arginine biosynthesis and catabolism have been linked to transforming growth factor-β (TGF-β)-induced activation of fibroblasts in the context of pulmonary fibrosis; however, a thorough study on the metabolic and signaling roles of arginine in the process of fibroblast activation has not been conducted. Here, we examined the role and regulation of arginine metabolism in lung fibroblasts activated with TGF-β. We found that TGF-β increases the expression of arginine biosynthetic and catabolic enzymes in lung fibroblasts. Surprisingly, using metabolic tracers of arginine and its precursors, we found little evidence of arginine synthesis or catabolism in lung fibroblasts treated with TGF-β. Despite this, arginine remained crucial for TGF-β-induced expression of collagen and α-smooth muscle actin. We found that arginine limitation leads to the activation of general control nonderepressible 2 (GCN2), while inhibiting TGF-β-induced mechanistic target of rapamycin complex 1 activation and collagen protein production. Extracellular citrulline could rescue the effect of arginine deprivation in an argininosuccinate synthase-dependent manner. Our findings suggest that the major role of arginine in lung fibroblasts is for charging of arginyl-tRNAs and promotion of signaling events which are required for fibroblast activation.

## Introduction

Idiopathic pulmonary fibrosis (IPF) is a fatal disease, with a median survival of 3.5 years and affecting approximately 150,000 people in the United States [1,2]. A defining feature of IPF is the transforming growth factor-β (TGF-β)-dependent activation of lung fibroblasts, leading to the excessive secretion of extracellular matrix proteins, including collagen [3-6]. Lung fibroblasts are the primary cells responsible for the structural remodeling and impairment of lung function characteristic of IPF and, thus, represent a key therapeutic target for the treatment of the disease [6-8]. Metabolic reprogramming has emerged as a key regulator of fibroblast activation and is increasingly studied as a target of therapeutic intervention for IPF [9-12]. We have previously demonstrated that *de novo* synthesis of glycine and proline, the two most abundant amino acids present in collagen protein, is critical for collagen protein synthesis downstream of TGF-β [13-15]. How the metabolism of other amino acids is regulated during fibrotic processes is poorly understood.

Arginine is a conditionally essential amino acid that has been linked with fibrotic processes; however, the role of arginine in lung fibroblasts is poorly understood [16]. Arginine is catabolized by arginase enzymes (ARG1, ARG2), producing ornithine, which is a precursor for proline and polyamines. In the liver, where the urea cycle is active, ornithine can be converted to citrulline, which is then converted to argininosuccinate by argininosuccinate synthase (ASS1) followed by resynthesis of arginine by argininosuccinate lyase (ASL). While the complete urea cycle is not present in all cells, citrulline can also be produced directly from arginine through the activity of nitric oxide synthases (NOS) and can be converted to arginine by ASS1 and ASL [17-21].

Both arginine synthesis and catabolism have been linked with lung fibrosis. ASS1 expression has been suggested to be reduced in IPF fibroblasts, causing these cells to be more dependent on extracellular arginine than control fibroblasts [22]. Arginine metabolites including ornithine, proline, and polyamines have been shown to be increased in the lungs of patients with IPF and in the fibrotic lungs of bleomycin-treated mice [23-25]. Arginase expression is increased in the lungs of bleomycin-treated mice and in lung fibroblasts and vascular smooth muscle cells (VSMCs) after treatment with TGF-β [26-28]. Chemical inhibition of arginase in lung fibroblasts has been shown to reduce collagen production after TGF-β, while inhibition of ornithine aminotransferase (OAT), the enzyme which converts ornithine to proline, inhibited TGF-β-induced collagen production in VSMCs and lung fibroblasts [26,28,29]. OAT expression correlates with lung function decline in IPF patients, and OAT inhibition was recently shown to inhibit the development of fibrosis downstream of bleomycin instillation in mice [23,29].

In addition to its central role in nitrogen metabolism, arginine is an important signaling regulator. We and others have demonstrated that the mechanistic target of rapamycin complex 1 (mTORC1) is a major regulator of amino acid homeostasis in lung fibroblasts [30,31]. mTORC1 integrates signals from extracellular signaling cascades and intracellular nutrients and is known to be particularly sensitive to cellular levels of arginine [32-35]. Arginine is also a negative regulator of the general control nonderepressible 2 (GCN2)-mediated branch of the integrated stress response, which responds to the accumulation of uncharged tRNAs, inhibiting protein translation [36].

Despite the continued interest in the role of arginine metabolism in fibrotic phenotypes, a thorough analysis of arginine metabolism in lung fibroblasts has not been conducted. Here, we used metabolic tracing and media dropout experiments to determine how TGF-β signaling regulates arginine biosynthesis and catabolism. When cells were cultured in Human Plasma-Like Medium (HPLM), which contains the arginine precursors ornithine and citrulline, we found little evidence of arginine synthesis or catabolism in lung fibroblasts. Almost all intracellular ornithine and citrulline came from extracellular stores. Extracellular ornithine was also the primary source of intracellular polyamines. When cells were cultured in DMEM, which lacks ornithine and citrulline, we found increased arginine catabolism by both NOS and arginase; however, surprisingly, glutamine, and not arginine, was the main source of cellular ornithine in these cells, contributing to proline and polyamine biosynthesis.

Although arginine was not significantly metabolized in lung fibroblasts, these cells maintained dependence on arginine for TGF-β-induced activation of mTORC1 and for collagen protein production. Addition of excess citrulline to the medium rescued the effect of arginine deprivation in an ASS1-dependent manner, suggesting that *de novo* biosynthesis can provide sufficient arginine in the absence of extracellular arginine. In total, our findings suggest that arginine catabolism is dispensable for lung fibroblasts and that the major role of arginine in lung fibroblasts is for charging of arginyl-tRNAs and for promoting a profibrotic signaling environment.

## Results

### Expression of arginine metabolic enzymes in normal and IPF fibroblasts

**Figure 1:**
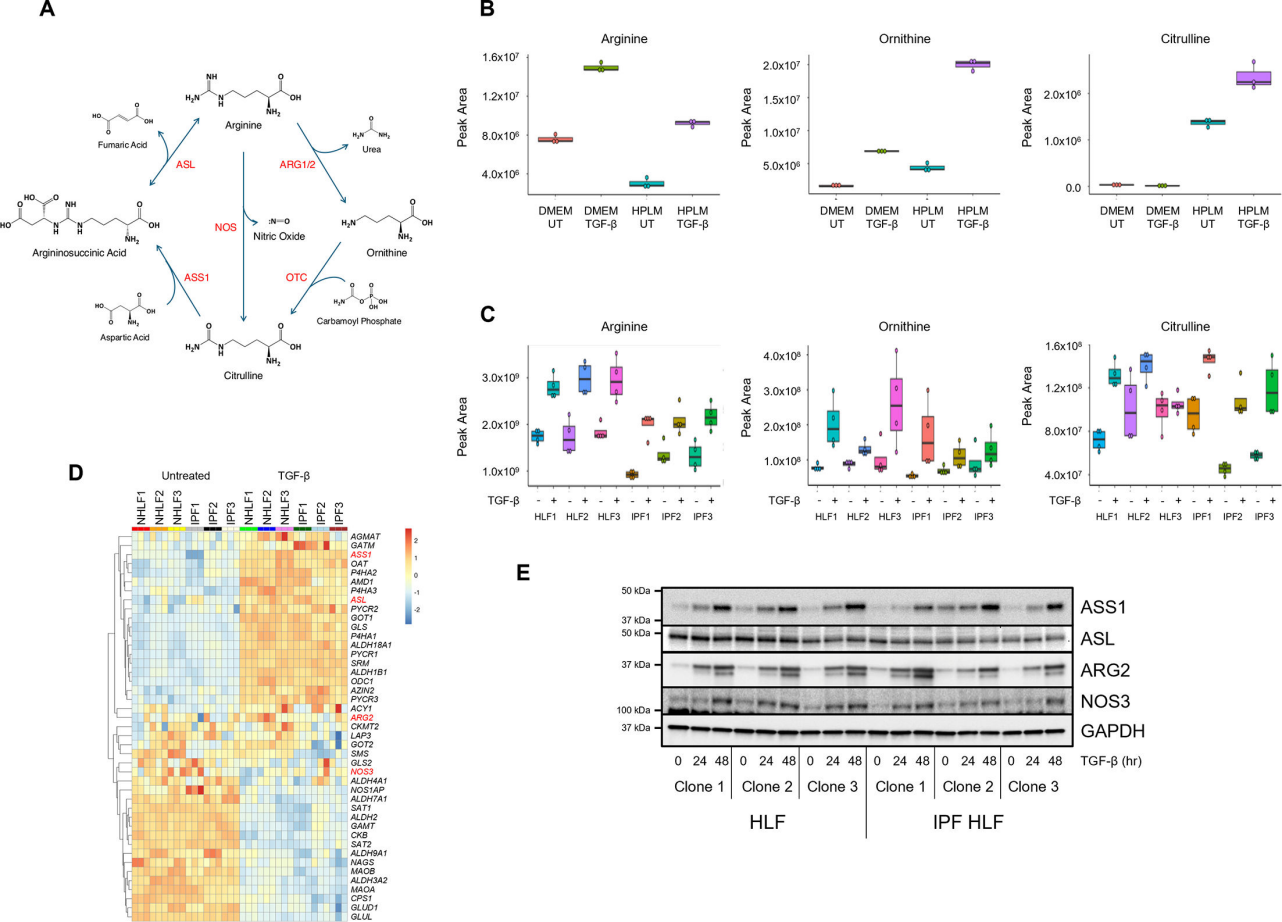
TGF-β increases expression of arginine metabolic enzymes and intracellular levels of arginine in human lung fibroblasts (HLFs). (**A**) Schematic illustration of arginine metabolism. Arginine is catabolized by arginase 1 and 2 (ARG1, ARG2) or by nitric oxide synthase (NOS), producing urea or nitric oxide, respectively. In cells with a functional urea cycle, ornithine is converted to citrulline by ornithine transcarbamylase. Citrulline is converted back to arginine through the combined actions of argininosuccinate synthase 1 (ASS1) and argininosuccinate lyase (ASL). (**B**) Intracellular levels of arginine, ornithine and citrulline in HLFs cultured in DMEM or Human Plasma-Like Medium (HPLM) and treated with TGF-β or left untreated for 48 hours. (**C**) Intracellular levels of arginine, ornithine, and citrulline from three clones of normal and three clones of IPF HLFs. Cells were cultured in HPLM and treated with TGF-β or left untreated for 48 hours. (**D**) Heatmap analysis showing the Kyoto Encyclopedia of Genes and Genomes (KEGG) arginine and proline metabolism pathway on RNA-seq data from three clones of normal and three clones of IPF HLFs (three biological replicates per clone). Cells were cultured in HPLM and treated with TGF-β or left untreated for 24 hours. (**E**) Western blot analysis showing protein expression of ASS1, ASL, ARG2, and NOS3 in three clones of normal and three clones of IPF HLFs. Cells were cultured in HPLM and treated with TGF-β for the indicated intervals.

It is poorly understood how fibroblast activation regulates arginine metabolism or how fibroblast activation depends on arginine biosynthetic or catabolic pathways (Figure 1A). Furthermore, most previous studies on arginine in fibroblasts have used conventional culture media such as DMEM, which contain high levels of arginine, but do not contain arginine precursors, including ornithine and citrulline. Therefore, we cultured human lung fibroblasts (HLFs) in either DMEM (0.4 mM arginine) or HPLM, (0.11 mM arginine, 0.07 mM ornithine, and 0.04 mM citrulline) in the presence or absence of TGF-β and extracted metabolites for analysis by liquid chromatography/mass spectrometry (LC/MS). We found that cellular levels of arginine were reduced when cells were cultured in HPLM, while intracellular levels of ornithine and citrulline were increased in HPLM (Figure 1B). While cells cultured in DMEM exhibited very low levels of intracellular citrulline, TGF-β treatment increased cellular levels of all three metabolites when cells were cultured in HPLM.

To gain a better understanding of the regulation of arginine metabolites in lung fibroblasts, we cultured three clones of control donor HLFs and three clones of IPF lung fibroblasts (IPF-HLF) in HPLM in the presence or absence of TGF-β (Figure 1C). TGF-β exposure increased cellular levels of arginine, ornithine, and citrulline in all six clones. Intracellular levels of ornithine and citrulline varied by clone; however, we observed that cellular arginine levels were consistently lower by 30% in IPF-HLFs compared with control HLFs.

To determine how arginine metabolic enzymes are affected by TGF-β, we performed RNA sequencing on RNA extracted from HLF and IPF-HLF cells and queried the KEGG arginine and proline metabolism pathway. As we have previously shown in Hamanaka et al. [15], TGF-β increased the expression of genes involved in the production of glutamate from glutamine and proline biosynthesis from glutamate (*GLS1*, *ALDH18A1*, *PYCR1*, *PYCR2*, and *PYCR3*) (Figure 1D). Genes involved in the metabolism of ornithine to proline (*OAT*) or to polyamines (*ODC1*, *SRM*) were also induced by TGF-β. Genes down-regulated by TGF-β included those involved in the reversal of these metabolic pathways, including catabolism of proline (*ALDH4A1*), catabolism of polyamines (*SAT1*, *SAT2*), and production of glutamine from glutamate (*GLUL*) (Figure 1D). Among the arginine metabolic enzymes, we found that *ASS1* mRNA exhibited the highest level of expression and the greatest inducibility by TGF-β (Figure 1D, Supplementary Figure S1). *ASL* mRNA was expressed at lower levels than ASS1 but was still induced by TGF-β (Figure 1D). *ARG2* and *NOS3* were expressed at low levels and were not significantly affected by TGF-β (Figure 1D, Supplementary Figure S1). Expression of *NOS1*, *NOS2*, and *ARG1* was not detected in the RNA-seq data. We did not find significant differences in arginine metabolic enzyme gene expression between normal and IPF fibroblasts (Supplementary Figure S1A).

We next used Western blot to analyze TGF-β-induced protein expression of arginine metabolic enzymes (Figure 1E). We found that TGF-β significantly induced the protein expression of ASS1 (Supplementary Figure S1B). While *ARG2* and *NOS3* mRNA expressions were not significantly affected by TGF-β, their proteins were induced in all clones. Conversely, while *ASL* mRNA was increased by TGF-β, its protein expression did not change. Differences in arginine metabolic enzyme protein expression between healthy and IPF fibroblasts were not detected (Supplementary Figure S1B).

**Figure 2:**
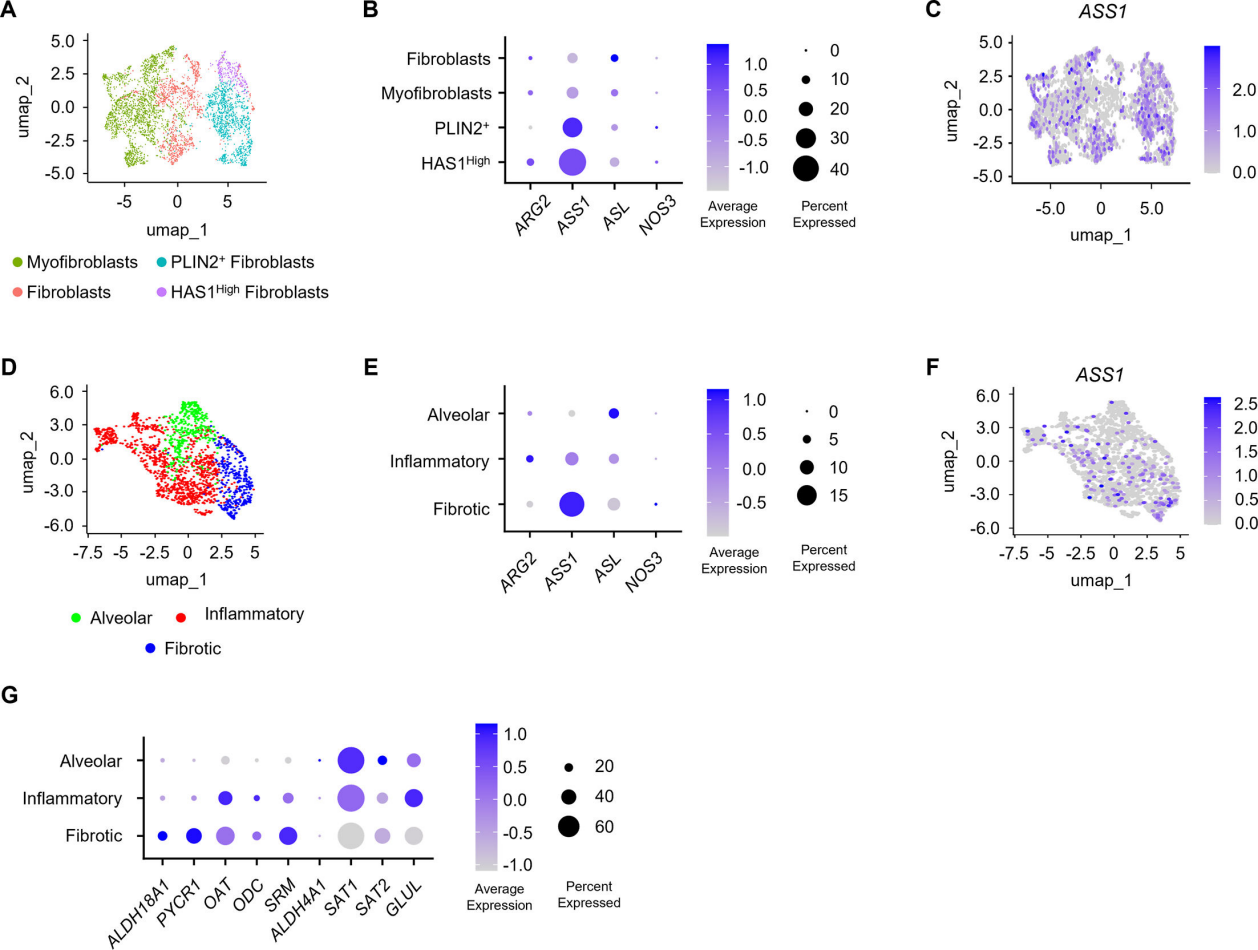
Single cell RNA-seq analysis of arginine metabolic enzymes in lung fibroblasts from patients with pulmonary fibrosis. (**A**) Uniform Manifold Approximation and Projection (UMAP) projection of the subclustering of lung fibroblast populations from patients with pulmonary fibrosis and control donor lungs as defined by Habermann et al. [37] showing fibroblast, myofibroblast, PLIN2^+^ fibroblast, and HAS1^High^ fibroblasts. (**B**) Dot plot representation of the expression of arginine metabolic enzymes in lung fibroblast populations as defined in (**A**). (**C**) UMAP projection of the expression of *ASS1* mRNA in lung fibroblasts from patient with pulmonary fibrosis and control donor lungs. (**D**) UMAP projection of the further subclustering of alveolar and myofibroblast populations from (**A**) as defined by Tsukui et al. showing alveolar fibroblasts, inflammatory fibroblasts, and fibrotic fibroblasts. (**E**) Dot plot representation of the expression of arginine metabolic enzymes in lung fibroblast populations as defined in (**D**). (**F**) UMAP projection of the expression of *ASS1* mRNA in lung fibroblast populations as defined in (**D**). (**G**) Dot plot representation of the expression of proline and polyamine biosynthetic and catabolic enzymes in lung fibroblast populations as defined in (**D**).

These findings demonstrate that profibrotic signaling promotes the expression of arginine metabolic enzymes in lung fibroblasts. To determine how these enzymes are expressed in fibroblast populations *in vivo*, we analyzed the expression of arginine metabolic enzymes in fibroblast populations from single cell RNA-seq data from patients with pulmonary fibrosis and control donors published previously by Habermann and colleagues [37]. Fibroblast populations identified by expression of *LUM*, *FBLN2*, and *PDGFRA* were subclustered into fibroblast, myofibroblast, *PLIN2* ^+^ fibroblasts, and *HAS1* ^High^ populations as defined by Habermann et al. [37] (Figure 2A). Lung fibroblasts from control subjects consist primarily of fibroblast and myofibroblast populations, while lung fibroblasts from patients with pulmonary fibrosis are characterized by expansion of the myofibroblast population and appearance of *PLIN2* ^+^ and *HAS1* ^High^ populations (Supplementary Figure S2A). These populations differ by gene expression signatures and localization within the lung [37]. We found that *ASS1* exhibited the highest level of expression among arginine metabolic enzymes in lung fibroblasts, with highest expression in the *PLIN2* ^+^ and *HAS1* ^High^ populations (Figure 2B, Supplementary Figure S2B). Expression of *ASS1* in fibroblast and myofibroblast populations was heterogeneous. In fibroblast populations, expression of *ASS1* was highest in *PI16*-expressing adventitial fibroblasts compared with alveolar fibroblasts (Figure 2C, Supplementary Figure S2C). In myofibroblasts, *ASS1* was expressed in *CTHRC1*-expressing fibrotic fibroblasts which express the highest level of *COL1A1* (Figure 2C, Supplementary Figure S2C).

Tsukui and colleagues recently demonstrated that alveolar fibroblasts give rise to inflammatory and fibrotic myofibroblast populations during fibrotic responses after lung injury [38]. Moreover, the development of fibrotic fibroblasts after lung injury is dependent on TGF-β signaling in alveolar fibroblasts [38]. Hence, we reclustered alveolar fibroblast and myofibroblast populations, as we have previously defined (Figure 2D, Supplementary Figure S3) [39]. Consistent with TGF-β-induced expression of *ASS1*, we found that the expression of *ASS1* was highest in fibrotic fibroblasts compared with alveolar and inflammatory fibroblast populations (Figure 2E and F). We also analyzed the expression of arginine and proline metabolic genes that were regulated by TGF-β treatment in Figure 1D. Consistent with the effects of TGF-β on cultured HLFs, we found that the expression of proline and ornithine metabolic enzymes elevated by TGF-β was increased in fibrotic fibroblasts compared with alveolar fibroblasts (Figure 2G). Similarly, proline and polyamine catabolic enzymes that were repressed by TGF-β exhibited greater expression in alveolar fibroblasts compared with fibrotic fibroblasts (Figure 1D). Together, our transcriptomic analysis from cultured HLFs and single-cell data suggests that TGF-β-dependent fibrotic processes promote the production of arginine, proline, and polyamines and repress the catabolism of proline and polyamines both *in vitro* and *in vivo*.

### Arginine is not significantly catabolized in HLFs

**Figure 3:**
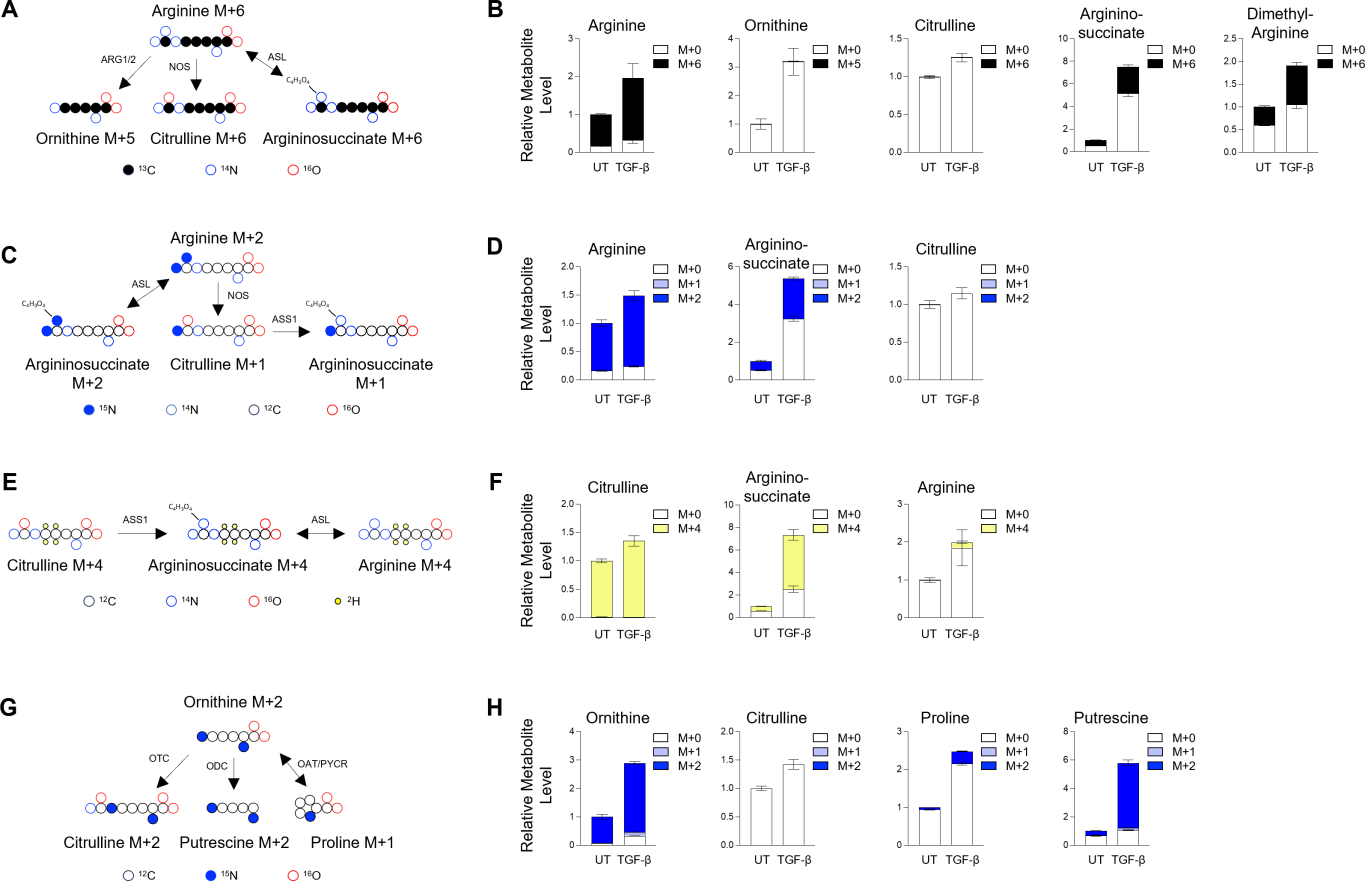
Metabolic tracing of arginine metabolism in human lung fibroblasts cultured in Human Plasma-Like Media (HPLM). (**A**) Schematic representation of the metabolism of ^13^C_6_ arginine. (**B**) Analysis of cellular arginine, ornithine, citrulline, argininosuccinate, and dimethylarginine in HLFs after labeling with ^13^C_6_ arginine HPLM in the presence or absence of TGF-β. (**C**) Schematic representation of the metabolism of guanido-^15^N_2_ arginine. (**D**) Analysis of cellular arginine, argininosuccinate, and citrulline in HLFs after labeling with guanido-^15^N_2_ arginine HPLM in the presence or absence of TGF-β. (**E**) Schematic representation of the metabolism of 4,4,5,5-D_4_ citrulline. (**F**) Analysis of cellular citrulline, argininosuccinate, and arginine in HLFs after labeling with 4,4,5,5-D_4_ citrulline HPLM in the presence or absence of TGF-β. (**G**) Schematic representation of the metabolism of ^15^N_2_ ornithine. (**H**) Analysis of cellular ornithine, citrulline, proline, and putrescine in HLFs after labeling with ^15^N_2_ ornithine HPLM in the presence or absence of TGF-β. Data are normalized to untreated group and presented as mean ± SEM from four biologic replicates.

Our findings suggest that arginine metabolism is regulated by fibrotic processes both *in vitro* and *in vivo*. To determine how lung fibroblasts utilize arginine, we formulated HPLM lacking arginine and substituted ^13^C_6_ arginine and traced arginine into downstream metabolites using LC/MS (Figure 3A). Strikingly, despite achieving over 80% labeling of intracellular arginine, we found that neither ornithine nor citrulline was significantly labeled downstream of arginine (Figure 3A, Supplementary Figure S4A). We did detect significant labeling in dimethylarginine (>40%) and in argininosuccinate (>30%). Dimethylarginine is the product of the hydrolysis of proteins in which arginine residues have been post-translationally methylated by protein arginine methyltransferases. This finding demonstrates the incorporation of labeled arginine into proteins with subsequent degradation. Argininosuccinate labeled on six carbons from ^13^C_6_ arginine can result from either reversal of the ASL reaction or from NOS-dependent citrulline production followed by conversion to argininosuccinate by ASS1. While low labeling on citrulline indicates that M + 6 argininosuccinate is the result of the reverse ASL reaction, to confirm, we labeled cells with guanido-^15^N_2_ arginine (Figure 3C). In this case, argininosuccinate produced by the reverse ASL reaction maintains both labeled nitrogens, while metabolism through NOS leads to the loss of one of the labeled nitrogen atoms. Consistent with the reverse ASL reaction, labeling on argininosuccinate from guanido-^15^N_2_ arginine was M + 2 (Figure 3D, Supplementary Figure S4B).

Because neither ornithine nor citrulline was significantly labeled downstream of arginine, we sought to determine if intracellular levels of these amino acids are determined primarily by their uptake from the extracellular space and whether they are metabolized following uptake. Therefore, we labeled HLFs in HPLM containing either ornithine labeled on both nitrogen atoms (^15^N_2_), or citrulline labeled with deuterium atoms on the four and five carbons (4,4,5,5-D_4_) (Figure 3E and G). Consistent with the low intracellular citrulline levels found in cells cultured in DMEM, which suggest that most intracellular citrulline is taken up from extracellular stores, we found that intracellular citrulline in HLFs comes almost 100% from extracellular citrulline (Figure 3F, Supplementary Figure S4C). Labeling from citrulline was found on argininosuccinate, demonstrating that most argininosuccinate in HLFs comes from exogenous citrulline. A small portion (<10%) of arginine was also labeled downstream of citrulline (Figure 3F, Supplementary Figure S4C). This finding suggests that the ASL reaction may function in either direction in HLFs depending on cellular conditions or metabolite concentrations.

Labeling HLFs with ornithine also showed that most intracellular ornithine comes from extracellular ornithine (Figure 3H, Supplementary Figure S4D). We did not detect any labeling from ornithine on citrulline, demonstrating that HLFs lack a complete urea cycle. Ornithine can also be used as a precursor for proline and polyamines. We found a small amount of labeling on proline, while the majority of the polyamine putrescine was labeled by extracellular ornithine (Figure 3H, Supplementary Figure S4D). These results suggest that a major role of extracellular ornithine in lung fibroblasts is polyamine production.

**Figure 4:**
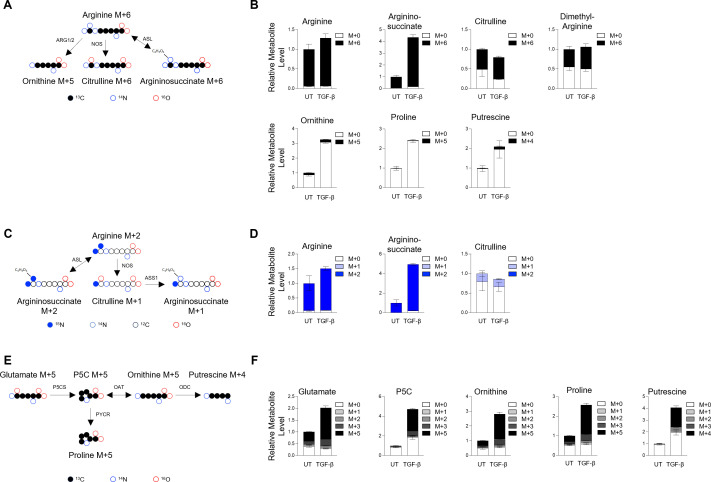
Metabolic tracing of arginine metabolism in human lung fibroblasts cultured in DMEM. (**A**) Schematic representation of the metabolism of ^13^C_6_ arginine. (**B**) Analysis of cellular arginine, argininosuccinate, citrulline, dimethylarginine, ornithine, proline, and putrescine in HLFs after labeling with ^13^C_6_ arginine DMEM in the presence or absence of TGF-β. (**C**) Schematic representation of the metabolism of guanido-^15^N_2_ arginine. (**D**) Analysis of cellular arginine, argininosuccinate, and citrulline in HLFs after labeling with guanido-^15^N_2_ arginine DMEM in the presence or absence of TGF-β. (**E**) Schematic representation of the metabolism of ^13^C_5_ glutamine. (**F**) Analysis of cellular glutamate, pyrroline-5-carboxylate, ornithine, proline, and putrescine in HLFs after labeling with ^13^C_5_ glutamine DMEM in the presence or absence of TGF-β. Data are normalized to untreated group and presented as mean ± SEM from four biologic replicates.

Because we were unable to detect arginine catabolism in HLFs cultured in HPLM, we considered the possibility that extracellular ornithine and citrulline inhibit the catabolism of arginine under these conditions. Thus, we cultured cells in DMEM containing ^13^C_6_ arginine to determine how arginine is metabolized in the absence of its catabolites extracellularly (Figure 4A). We found that culturing cells in DMEM resulted in the majority of citrulline being labeled on six carbons, indicating that the low amount of citrulline in DMEM-cultured HLF comes from nitric oxide synthesis (Figure 4B, Supplementary Figure S5A). Surprisingly, only a small amount (<15%) of ornithine and downstream putrescine was labeled from arginine, and no labeling was found on proline, indicating a low amount of arginase activity (Figure 4B, Supplementary Figure S5A). We also found that argininosuccinate was almost completely labeled M + 6 by ^13^C_6_ arginine (Figure 4B, Supplementary Figure S5A). Labeling with guanido-^15^N_2_ arginine demonstrated that this occurred through the reverse ASL reaction (Figure 4D, Supplementary Figure S5B).

Arginine is predicted to be the major source of cellular ornithine and polyamines in DMEM-cultured cells; thus, it was surprising that the majority of these molecules were unlabeled after culture in ^13^C_6_ arginine. OAT produces pyrroline-5-carboxylate (P5C), a precursor for proline, in a reversible reaction (Figure 4E). Glutamate is also a precursor for P5C through the action of P5C synthase. We sought to determine whether glutamate is a major source of ornithine and polyamines through the reverse OAT reaction, producing ornithine from glutamate-derived P5C. We labeled HLFs with ^13^C_5_ glutamine (Figure 4E). We found that this resulted in the majority of intracellular glutamate being labeled through the glutaminase reaction (Figure 4F, Supplementary Figure S5C). As we have previously demonstrated in Hamanaka et al. [15], glutamine is an important source of P5C and proline in HLFs (Figure 4F, Supplementary Figure S5C). We also found significant labeling from glutamine on ornithine and putrescine, demonstrating that glutamine, and not arginine, is the major source of these metabolites in HLFs cultured in DMEM (Figure 4F, Supplementary Figure S5C).

### Arginine is required for TGF-β-induced signaling and collagen production

**Figure 5:**
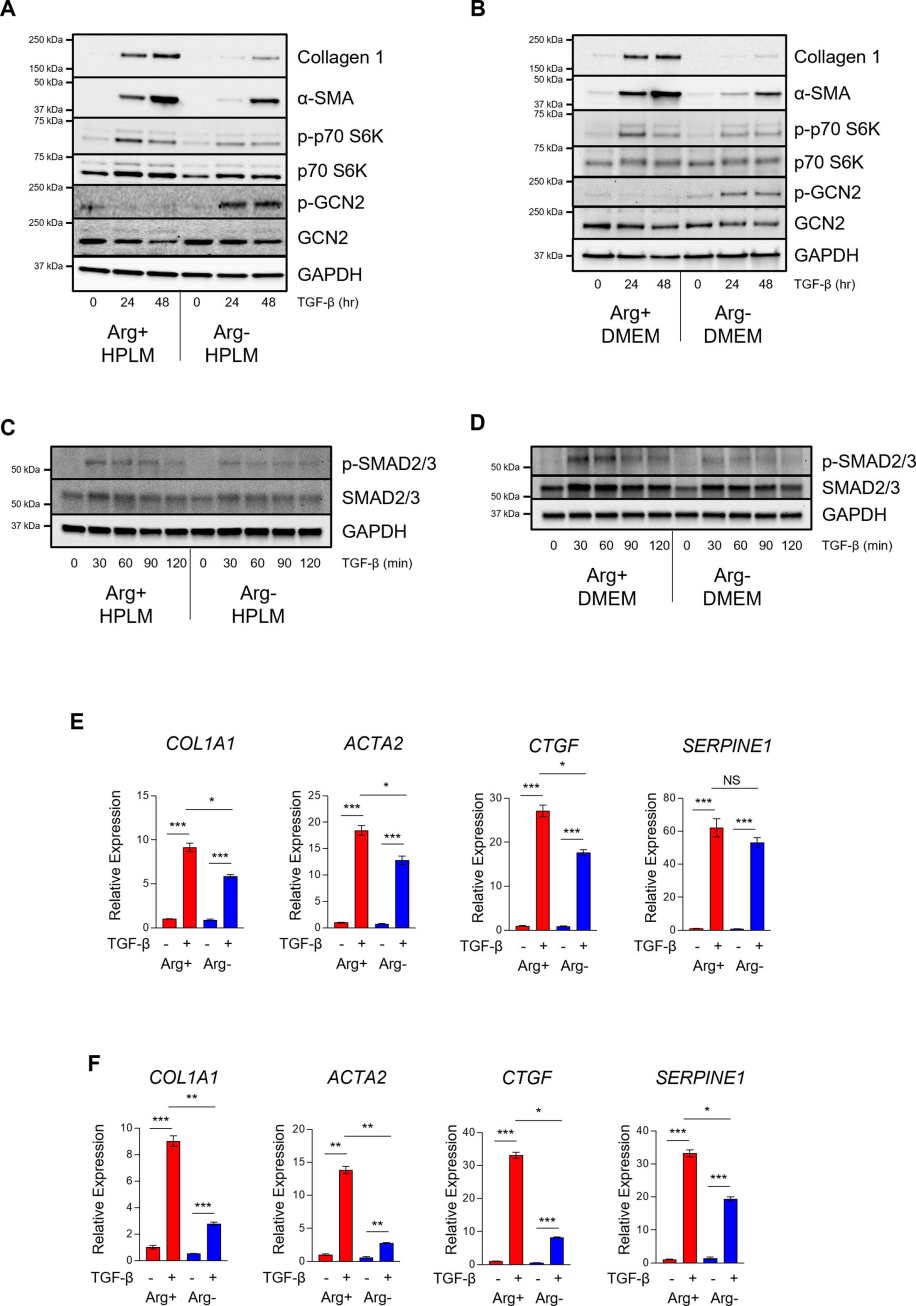
Arginine is required for TGF-β-induced signaling and gene expression in human lung fibroblasts (HLFs). (**A–B**) Western blot analysis of collagen 1 and α-smooth muscle actin protein expression and S6-kinase and GCN2 phosphorylation in HLFs cultured in either (**A**) Human Plasma-Like Medium (HPLM) or (**B**) DMEM containing or lacking arginine. Cells were treated with TGF-β for the indicated intervals. (**C–D**) Western blot analysis of SMAD2/3 phosphorylation in HLFs cultured in either (**C**) HPLM or (**D**) DMEM containing or lacking arginine. Cells were treated with TGF-β for the indicated intervals. (**E–F**) qPCR analysis of *COL1A1*, *ACTA2*, *CTGF*, and *SERPINE1* mRNA expression in HLFs cultured in the either (**E**) HPLM or (**F**) DMEM containing or lacking arginine. Cells were treated with TGF-β for 24 hours or left untreated. Data are normalized to Arg+ and no TGF-β treatment (Arg+/TGF-β-) group and presented as mean ± SEM from four biologic replicates. *P<0.05, **P<0.01, ***P<0.001 by two-way Analysis of Variance (ANOVA) using Tukey’s post-test.

Our findings demonstrate that arginine is not significantly catabolized in HLFs and that the downstream products of arginine catabolism, ornithine and citrulline, are largely taken up from the extracellular space. Thus, we sought to determine whether arginine itself is dispensable for fibroblast activation. We cultured HLFs in either DMEM or HPLM containing standard arginine concentrations or no arginine and treated with TGF-β (Figure 5A and B). We found that arginine deficiency inhibited TGF-β-induced induction of α-smooth muscle actin (α-SMA) and greatly reduced the induction of collagen protein in cells cultured in either DMEM or HPLM. This was associated with reduced phosphorylation of the mTOR target S6-kinase, consistent with the role of arginine as an mTOR activator. We also observed phosphorylation of GCN2, suggesting that arginine deprivation leads to the accumulation of uncharged arginyl-tRNAs in HLFs (Figure 5A and B). To determine if arginine deficiency regulated canonical signaling downstream of TGF-β, we measured phosphorylation of mothers against decapentaplegic homolog 2/3 (SMAD2/3) in the presence or absence of arginine. Consistent with reduced fibroblast activation in the absence of arginine, we found that phosphorylation of SMAD2/3 was attenuated in HLFs cultured in either DMEM or HPLM (Figure 5C and D). We also examined the expression of the TGF-β-induced mRNAs *COL1A1*, *ACTA2*, *CTGF*, and *SERPINE1* in arginine-deficient cells. Consistent with reduced SMAD2/3 phosphorylation downstream of TGF-β, the expression of these mRNAs was also reduced in cells lacking arginine (Figure 5E and F). TGF-β-induced transcription was less sensitive to arginine deprivation when cells were cultured in HPLM compared with cells cultured in DMEM, suggesting that the effect of arginine deficiency may be reduced in the presence of arginine precursors.

To determine whether the effects of arginine depletion were the result of down-regulation of the TGF-β receptor, we measured the expression of TGF-β receptor I and II (TβR1 and TβR2). We found that the two receptor subunits displayed different expression patterns after exposure of HLFs to TGF-β. TβR1 expression was increased at 24 and 48 hours after TGF-β exposure (Supplementary Figure S6A and B). TβR2 expression decreased at 24 hours and returned at 48 hours after TGF-β exposure (Supplementary Figure S6A and B). Expression of both receptor subunits was similar at baseline between arginine-deficient and arginine-replete cells; however, TβR1 induction at 24 and 48 hours was reduced in arginine-deficient cells, and the return of TβR2 expression at 48 hours did not occur in arginine-deficient cells (Supplementary Figure S6A and B). Reduced TGF-β receptor induction in arginine-deficient conditions was concomitant with the activation of GCN2 (Figure 5A and B).

### Exogenous citrulline and ASS1 can rescue fibroblast activation in arginine-deprived HLFs

**Figure 6:**
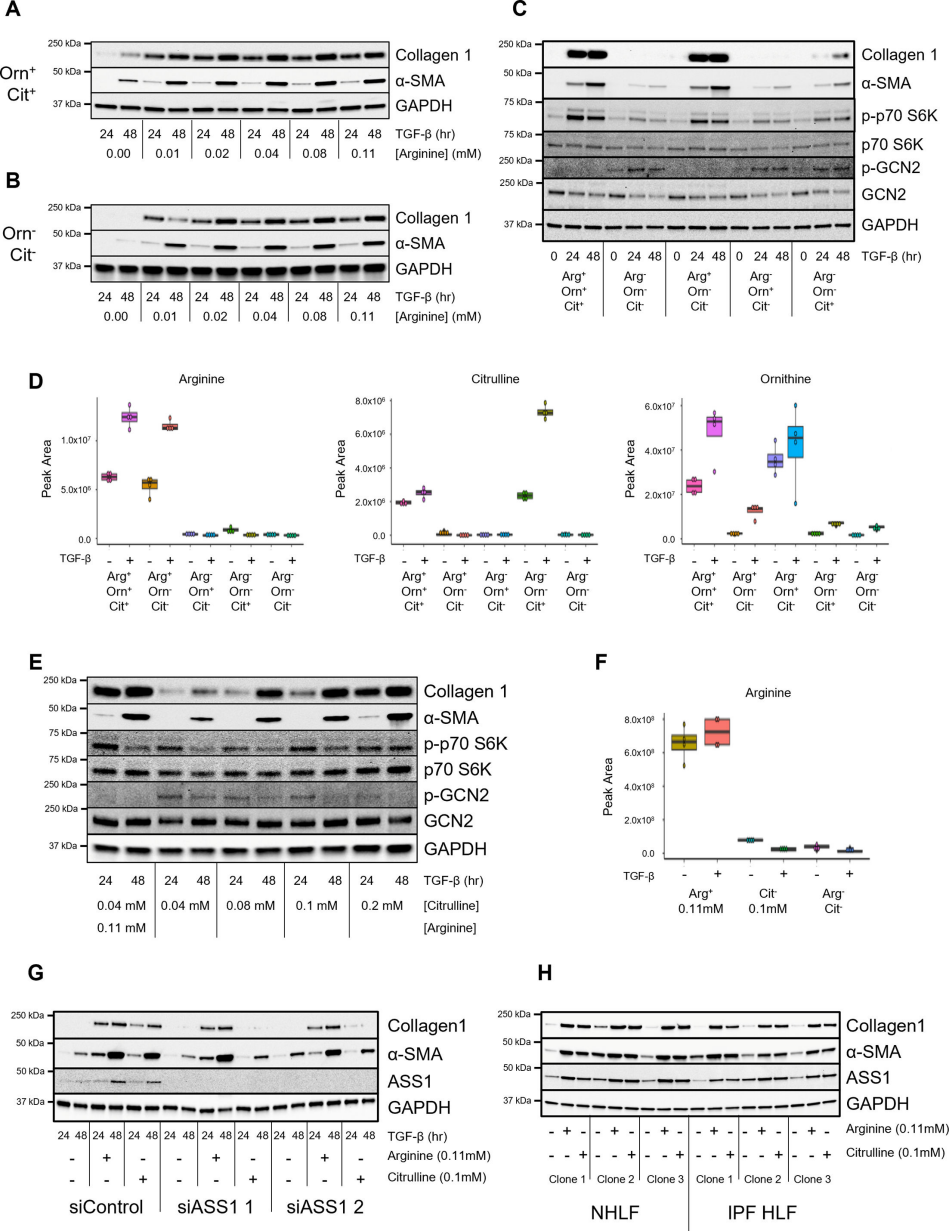
Metabolism of extracellular citrulline through ASS1 can rescue the effect of arginine depletion in human lung fibroblasts (HLFs). (**A–B**) Western blot analysis of collagen 1 and α-smooth muscle actin (α-SMA) protein expression in HLFs cultured in Human Plasma-Like Medium (HPLM) either (**A**) containing ornithine and citrulline, or (**B**) lacking ornithine and citrulline. Cells were treated with TGF-β for the indicated intervals in the presence of the indicated concentrations of arginine. (**C**) Western blot analysis of collagen 1 and α-smooth muscle actin protein expression and S6-kinase and GCN2 phosphorylation in HLFs cultured in HPLM that (1) contains arginine, ornithine, and citrulline; (2) contains no arginine, ornithine, or citrulline; (3) contains just arginine; (4) contains just ornithine; (5) contains just citrulline. Cells were treated with TGF-β for the indicated intervals. (**D**) Intracellular levels of arginine, ornithine, and citrulline from cells cultured in HPLM as in (**C**). Cells were treated with TGF-β or left untreated for 48 hours. (**E**) Western blot analysis of collagen 1 and α-SMA protein expression and S6-kinase and GCN2 phosphorylation in HLFs cultured in HPLM containing no ornithine or arginine, with the indicated concentrations of citrulline. Cells were treated with TGF-β for the indicated intervals. (**F**) Intracellular levels of arginine from cells cultured in HPLM as in (**E**). Cells were treated with TGF-β or left untreated for 48 hours. (**G**) Western blot analysis of collagen 1 and α-SMA protein expression in control and *ASS1* knockdown HLFs cultured in HPLM that contains arginine (0.11 mM) and citrulline (0.1 mM) as indicated. Cells were treated with TGF-β for the indicated intervals. (**H**) Western blot analysis of collagen 1 and α-SMA protein expression in HLFs cultured in HPLM that contains arginine (0.11 mM) and citrulline (0.1 mM) as indicated. Cells were treated with TGF-β for 48 hours.

Our results suggest that *de novo* arginine synthesis can ameliorate the effects of arginine deficiency in TGF-β-treated fibroblasts. Indeed, we observed that when HLFs were cultured in HPLM containing ornithine and citrulline, extracellular arginine concentrations could be reduced to, as low as, 0.01 mM, and TGF-β-mediated induction of collagen and α-SMA protein expression was unaffected. At 0.0 mM arginine, induction of collagen and α-SMA protein was still observed (Figure 6A). When cells were cultured in media lacking ornithine and citrulline, arginine concentrations could only be reduced to 0.02 mM before the expression of collagen and α-SMA protein was inhibited (Figure 6B). Collagen production was reduced at 0.01 mM arginine and unobservable at 0.0 mM arginine.

To determine how extracellular ornithine and citrulline affect TGF-β-induced protein production in cells lacking arginine, we formulated HPLM lacking arginine, ornithine, and citrulline, and then added back each amino acid individually to assess their role in promoting fibroblast activation downstream of TGF-β. We found that in the absence of all three amino acids (Arg^−^Orn^−^Cit^−^), TGF-β-induced production of collagen and α-SMA was abolished (Figure 6C). Addition of arginine to this media (Arg^+^Orn^−^Cit^−^) resulted in a complete rescue of collagen and α-SMA protein expression. This was associated with increased S6-kinase phosphorylation and loss of GCN2 phosphorylation. Addition of citrulline (Arg^−^Orn^−^Cit^+^), but not ornithine (Arg^−^Orn^+^Cit^−^), partially rescued the effect of arginine deficiency. These findings suggest that while ornithine and citrulline do not play a major role in supporting TGF-β-induced activation of lung fibroblasts when arginine is present, citrulline, but not ornithine, can be used as a substrate to promote fibroblast activation in the absence of arginine. When we examined intracellular levels of arginine metabolites in HLFs cultured in these media, we found that arginine levels were similar between Arg^+^Orn^+^Cit^+^ cultured cells and Arg^+^Orn^−^Cit^−^ cultured cells, suggesting that minimal arginine synthesis occurs when extracellular arginine is present (Figure 6D). Intracellular ornithine levels were highest in cells cultured in the presence of extracellular ornithine; however, extracellular arginine (Arg^+^Orn^−^Cit^−^) could partially rescue ornithine levels (Figure 6D). Citrulline levels were similar at baseline between Arg^+^Orn^+^Cit^+^ cultured cells and Arg^−^Orn^−^Cit^+^; however, intracellular citrulline was highest after TGF-β in cells cultured in only citrulline, suggesting that citrulline uptake is increased in the absence of arginine (Figure 6D). Extracellular citrulline but not ornithine increased arginine levels at baseline (Figure 6D, Supplementary Figure S7); however, after treatment with TGF-β, intracellular levels of arginine were not significantly different from cells cultured in Arg^−^Orn^−^Cit^−^ media, suggesting that TGF-β increases arginine usage and that 0.04 mM extracellular citrulline cannot contribute to arginine accumulation in the absence of extracellular arginine.

We sought to determine whether increasing extracellular citrulline could rescue fibroblast activation in the absence of arginine by titrating extracellular citrulline levels in medium lacking arginine and ornithine. We found that increasing extracellular citrulline concentrations to 0.1 mM rescued TGF-β-induced collagen and α-SMA protein production (Figure 6E). This was associated with partial rescue of TGF-β receptor expression (Supplementary Figure S6C). In addtion, GCN2 phosphorylation was reduced on day 2 of TGF-β treatment. Despite this rescue of TGF-β-induced fibroblast activation, we found that intracellular arginine levels remained very low in cells cultured in 0.1 mM extracellular citrulline (Figure 6F). This suggests that while arginine levels were extremely low in the absence of extracellular arginine, *de novo* arginine production was sufficient to maintain the signaling roles of arginine, allowing collagen induction downstream of TGF-β. To test this hypothesis, we knocked down ASS1 expression using siRNA and treated cells with TGF-β in the presence of Arg^−^Orn^−^Cit^−^ media supplemented with either 0.11 mM arginine, 0.1 mM citrulline, or no amino acid. Consistent with a role for ASS1 in promoting arginine synthesis in the absence of extracellular arginine, we found that ASS1 was not required for fibroblast activation when extracellular arginine was present (Figure 6G). However, when cells were cultured in 0.1 mM citrulline with no arginine, ASS1 became required to support collagen and α-SMA protein production.

Because IPF fibroblasts have been suggested to be deficient in arginine biosynthesis [22], we sought to determine whether IPF HLFs display greater sensitivity to arginine deprivation than normal HLFs. We cultured control and IPF HLFs in HPLM containing either arginine (0.11 mM), citrulline (0.1 mM), or none of these amino acids, and treated the cells with TGF-β for 48 hours. We found that IPF HLFs were capable of inducing collagen and α-SMA protein in medium containing only citrulline, suggesting that IPF HLFs have no defect in their ability to synthesize arginine (Figure 6H).

## Discussion

Metabolic reprogramming in lung fibroblasts is required to support matrix protein production in the context of pulmonary fibrosis [9-12]. Arginine and its metabolism have been linked with matrix production in lung fibroblasts; however, a thorough analysis of the role of arginine biosynthesis and catabolism has not previously been conducted. Here, our work suggests that the major roles of arginine in lung fibroblasts are to support the production of arginine-containing proteins, to signal amino acid sufficiency to mTOR and GCN2, and to maintain TGF-β-dependent signaling.

We found that the proteins of arginine metabolic enzymes ASS1, ARG2, and NOS3 were induced by TGF-β in lung fibroblasts *in vitro*. Interestingly, mRNA expression of *ASL*, *ARG2*, and *NOS3* did not correlate with their protein expression. These findings are consistent with recent work on translational responses to TGF-β showing that many TGF-β-induced proteins are regulated at the level of protein translation [40-42]. Surprisingly, despite the up-regulation of ARG2 and NOS3 proteins by TGF-β, we found that catabolism of arginine does not occur to a significant extent in HLFs. When HLFs were cultured in medium containing citrulline and ornithine, we found that intracellular levels of these amino acids were almost completely derived from extracellular stores, and not production from arginine. When cells were cultured in the absence of citrulline and ornithine, we found evidence that both arginase and NOS-dependent pathways are active. While it is not possible to determine from our data if either pathway is preferentially used by HLFs, it is striking that the minority of cellular ornithine comes from arginase activity when HLFs are cultured in DMEM. We and others have previously demonstrated that glutamine-dependent proline synthesis is required to support collagen production downstream of TGF-β [15,43]. Ornithine has been hypothesized to be a precursor for proline through the activity of OAT; however, our findings suggest that OAT activity proceeds primarily in the direction of ornithine synthesis even in cells cultured in DMEM, which contains abundant arginine and no proline. OAT expression has been shown to be elevated in IPF lung tissue and expressed in fibroblasts. Inhibition of OAT reduces collagen protein production downstream of TGF-β [23,29]. Our results suggest that this may be more related to the production of ornithine and downstream production of polyamines than to the production of proline. It is also possible that a nonmetabolic role for OAT exists.

Our findings also suggest that the ASL reaction in HLFs proceeds primarily in the direction of argininosuccinate synthesis from arginine. The ASL reaction is known to be reversible and is a net producer of argininosuccinate in certain conditions such as in fumarate hydratase-deficient cancers, in which the consumption of fumarate by ASL enables survival [44]. Cancer cell lines have been shown to exhibit reverse ASL activity when cultured in DMEM, but this was abolished when cells were cultured in a physiologic medium containing citrulline and low arginine [45]. We found that HLFs still exhibit significant reverse ASL activity even in HPLM. Whether this reversal of ASL and the maintenance of cellular argininosuccinate pools plays an important role in lung fibroblasts remains to be determined.

Our results show that in the absence of extracellular citrulline, most intracellular citrulline comes from arginine metabolism through NOS. Little is known about the role of NOS in lung fibroblasts or in IPF. Alveolar NO concentrations are elevated in IPF patients [46]; however, it is not known what cell types are the main contributor to this elevation. NOS2 expression is increased when lung fibroblasts are induced to proliferate, and NOS inhibition reduced fibroblast growth rate [47]. Some evidence suggests that NO production in fibroblasts promotes collagen synthesis [48-50]; however, NOS triple knockout mice (lacking all three NOS isoforms) exhibit increased severity of pulmonary fibrosis after instillation of bleomycin [51]. Our transcriptomic findings suggest that NOS3 is the primary NOS expressed in lung fibroblasts both *in vitro* and *in vivo*; however, our metabolic labeling experiments suggest that it does not play a major role in arginine metabolism.

ASS1 has been suggested to be reduced in IPF HLFs, increasing their sensitivity to arginine depletion. Our findings show that ASS1 is required to support collagen production when HLFs are cultured in the absence of arginine. We found no evidence that ASS1 was limiting in IPF HLFs as these cells could produce collagen when cultured using citrulline as a precursor for arginine production. Our findings also demonstrate that HLFs are able to produce collagen and α-SMA protein even after reducing extracellular arginine levels to one-fifth of what is found in human plasma. Furthermore, while citrulline-dependent arginine production is sufficient to promote collagen and α-SMA production, cells cultured in citrulline maintain remarkably low intracellular arginine levels. Thus, it is unlikely that arginine becomes limiting for HLFs in the context of fibrosis. We did note that arginine concentrations were reduced in IPF HLFs. Whether this is due to increased arginine catabolism, reduced arginine uptake, or reduced breakdown of arginine-containing proteins will need to be explored further. Furthermore, our transcriptomic work shows that *ASS1* is highly expressed in pathologic fibroblast populations from pulmonary fibrosis patients. We found that the expression of *ASS1,* as well as enzymes involved with the production of proline and polyamines, are elevated in fibrotic fibroblasts compared with alveolar fibroblasts. While the etiology of *PLIN2* ^+^ and *HAS1* ^High^ fibroblasts defined by Habermann et al. has not been defined, our findings show that these disease-associated cells also exhibit high levels of *ASS1* expression.

Our work suggests that HLFs do not significantly metabolize arginine and that intracellular levels of arginine above a threshold required to activate mTORC1 and prevent GCN2 activation are sufficient to support collagen protein production. Surprisingly, we found that arginine depletion led to reduced SMAD phosphorylation and SMAD-dependent transcription downstream of TGF-β. We have previously shown that SMAD phosphorylation occurs independently of PI3K and mTOR signaling [30]. Our findings suggest that GCN2 may feed back onto the TGF-β pathway, potentially through inhibiting the expression of the TGF-β receptor. Thus, GCN2 may be an inhibitor of profibrotic signaling pathways under amino acid-deficient conditions.

In conclusion, we find that arginine is a major regulator of TGF-β-induced activation of lung fibroblasts. While arginine biosynthesis and catabolism can be increased depending on local nutrient conditions, our findings suggest that the major requirements for arginine are for the production of arginine-containing proteins and for its role as a signaling molecule. Our findings suggest that therapies that target the signaling role of arginine may prevent collagen production by activated tissue fibroblasts in IPF and in other fibrotic diseases.

## Methods

### Lung fibroblast culture

Normal HLFs and IPF lung fibroblasts (Lonza) were cultured in FGM2 (PromoCell) as previously described in Nigdelioglu et al. [13]. Cells were serum starved in DMEM (Gibco) containing 0.1% bovine serum albumin (BSA), 5.5 mM glucose, 2 mM glutamine, and 1 mM pyruvate for 24 hours prior to treatment with TGF-β (1 ng/ml, Peprotech). For arginine starvation in DMEM, SILAC DMEM Flex Media (Gibco) was supplemented with 5.5 mM glucose, 2 mM glutamine, 1 mM pyruvate, and 0.8 mM lysine hydrochloride and 0.1% BSA. Arginine was added to control media at 0.4 mM. For experiments with HPLM, media were formulated according to the original recipe protocol of Cantor et al. [52], with the exception of arginine, citrulline, and ornithine. These were dissolved in Arg^−^Orn^−^Cit^−^ HPLM as 10× stocks and added to the parental media as indicated. Labeled metabolites were purchased from Cambridge Isotope Laboratories. ^13^C_6_ Arginine (CLM-2265), Guanido ^15^N_2_ Arginine (NLM-395), ^15^N_2_ Ornithine (NLM-3610), 4,4,5,5-D_4_ Citrulline (DLM-6039), and ^13^C_5_ Glutamine (CLM-1822).

### siRNA knockdowns

For siRNA knockdowns, 1 × 10^6^ HLFs were transfected with 250 pmol ON-Silencer Select siRNA (Ambion). Cells were plated on 10-cm dishes for 24 hours and then replated for experiments as above. Ambion product numbers are nontargeting siRNA- 4390843, siASS1 1- S1684, and siASS1 2- S1685.

### Western blotting

Cells were lysed, and electrophoresis was performed as we previously described in Hamanaka and Mutlu [53]. Wells were lysed in 100-μl Urea Sample Buffer (8M deionized urea, 1% SDS, 10% Glycerol, 60 mM Tris pH 6.8, 0.1% pyronin-Y, 5% β-mercaptoethanol). Lysates were run through a 28-gauge needle and were electrophoresed on Criterion gels (Bio-Rad) and transferred to nitrocellulose using a Trans-Blot Turbo (Bio-Rad) set to the mixed MW program. Primary antibodies used were as follows: Collagen 1 (Abcam, ab138492), α-SMA (Sigma, A2547), Phospho-GCN2 (Cell Signaling, 94668), GCN2 (Cell Signaling, 3302), Phospho-P70S6K (Cell Signaling, 9234), P70S6K (Cell Signaling, 9202), Phospho-SMAD2/3 (Cell Signaling, 9520), SMAD2/3 (Cell Signaling, 9523), ASS1 (Invitrogen, PA-5-82740), ASL (Novus, NBP1-87462), NOS3 (Proteintech, 27120-1-AP), ARG2 (Invitrogen PA5-78820), TβR1 (Cell Signaling, 49728), TβR2 (Cell Signaling, 41896), and glyceraldehyde-3-phosphate dehydrogenase (GAPDH) (Cell Signaling, 2118).

### RNA isolation and quantitative PCR

RNA was isolated using the GenElute Total RNA Purification Kit (Sigma) and was reverse-transcribed using iScript Reverse Transcription Supermix (Bio-Rad). Quantitative mRNA expression was determined by quantitative reverse transcription polymerase chain reaction (qRT-PCR) using ITaq Universal SYBR Green Supermix (Bio-Rad). Primers used for PCR were as follows: *COL1A1* (F:5′-GGTCAGATGGGCCCCCG-3′, R:5′-GCACCATCATTTCCACGAGC-3′), *ACTA2* (F:5′-GGCGGTGCTGTCTCTCTAT-3′, R:5′-CCAGATCCAGACGCATGATG-3′), *CTGF* (F:5′-GGCTTACCGACTGGAAGAC-3′, R:5′-AGGAGGCGTTGTCATTGG-3′), *SERPINE1* (F:5′-GGCTGACTTCACGAGTCTTTC-3′, R:5′-GCGGGCTGAGACTATGACA-3′).

### RNA-seq analysis

Total RNA isolated as above was sequenced on an Illumina NovaSEQ 6000 at the University of Chicago Genomics Core Facility (100 bp paired end). Sequence qualities of generated FASTQ files were assessed using FastQC. Transcript expression was quantified using Kallisto v.0.46.1 [54]. The Kallisto index was created with the GENCODE (Human Release 45, GRCh38.14). Quantification was performed in quant mode using default parameters. Gene abundances were computed using the R package tximport v.1.30.0 [55]. Differential gene expression analysis was performed using the edgeR package v.4.0.16 quasi-likelihood F model [56]. Differential gene expression was considered significant for genes with an FDR-adjusted *P*-value ≤ 0.05. All packages were run in RStudio (2023.06.2+561) with R version 4.3.1.

### Liquid chromatography mass spectrometry

After treatment as above, cells were quenched with 1 ml of dry ice cold 80% methanol and stored at −80°C until analysis. On the day of the analysis, samples were sonicated for 3 minutes in ice cold water-filled sonicator followed by 5 minutes of mixing with the thermomixer at 2000 rpm and 4°C, 20 minutes incubation on ice and centrifuge at 18,000 g and 4°C for 20 minutes. The supernatant was dried down using the Genevac EZ-2.4 elite evaporator. The dried samples were resuspended in ice-cold 60/40 acetonitrile/water before LC-MS analysis. All solvents were LC-MS grade and obtained from Fisher Scientific.

Metabolites separation and detection were performed as described in Apiz Saab et al. [57]. In brief, the Thermo Scientific Vanquish Horizon UHPLC system and Atlantis BEH Z-HILIC (2.1 × 150 mm, 2.5 µM; part # 186009990; Waters Corporation) column at acidic pH or iHILIC-(P) Classic (2.1 × 150 mm, 5 µm; part # 160.152.0520; HILICON AB) column at basic pH was used to detect the urea cycle-related metabolites. For the acidic pH method, the mobile phase A (MPA) was 10 mM ammonium formate containing 0.2% formic acid, and mobile phase B (MPB) was acetonitrile containing 0.1% formic acid. The column temperature, injection volume, and flow rate were 30°C, 5 µl, and 0.2 ml/minute, respectively. The chromatographic gradient was 0 minute: 90% B, 15 minutes: 20% B, 16 minutes: 20% B, 16.5 minutes: 90% B, 17 minutes: 90% B, and 23 minutes: 90% B. The flow rate was increased to 0.4 ml/minute for 4.7 minutes during the re-equilibration. MS detection was done using Orbitrap IQ-X Tribrid mass spectrometer (Thermo Scientific) with a heated-electrospray ionization (H-ESI) probe operating in switch polarity mode for both methods, except the *in-vitro* ^13^C_5_ citrulline tracing experiment data were collected only in positive mode. MS parameters were as follows: spray voltage: 3800 V for positive ionization and 2500 V for negative ionization modes, sheath gas: 80, auxiliary gas: 25, sweep gas: 1, ion transfer tube temperature: 300°C, vaporizer temperature: 300°C, automatic gain control target: 25%, and a maximum injection time of 80 milliseconds (ms). For the basic pH method, MPA was 20 mM ammonium bicarbonate at pH 9.6, adjusted by ammonium hydroxide addition, and MPB was acetonitrile. The column temperature, injection volume, and the flow rate were 40°C, 2 µl, and 0.2 ml/minute, respectively. The chromatographic gradient was 0 minute: 85% B, 0.5 minute: 85% B, 18 minutes: 20% B, 20 minutes: 20% B, 20.5 minutes: 85% B and 28 minutes: 85% B. MS parameters were as follows: spray voltage: 3600 V for positive ionization and 2800 for negative ionization modes, sheath gas: 35, auxiliary gas: 5, sweep gas: 1, ion transfer tube temperature: 250°C, vaporizer temperature: 350°C, AGC target: 100%, and a maximum injection time of 118 ms.

For both methods, data acquisition was done using the Xcalibur software (Thermo Scientific) in full-scan mode with a range of 70–1000 m/z at 120K resolution (acidic pH) and 60K (basic pH). Metabolite identification was done by matching the retention time and MS/MS fragmentation to the reference standards. Data analysis was performed using Thermo Scientific Tracefinder 5.1, Compound Discoverer 3.3 software, and natural abundance correction was performed using the IsoCor [58].

### Analysis of single cell RNA-seq datasets

Preprocessed data (aligned and UMI-quantified) data from non-fibrotic control and pulmonary fibrosis lungs data (GSE135893), from Habermann et al. [37] were downloaded from GitHub (https://github.com/tgen/banovichlab/tree/master/pulmonary_fibrosis/10x_scRNA-Seq_2019). Dimensionality reduction, clustering, and visualization of the gene-cell count matrix of the single cell RNA-seq data were processed and analyzed using the Seurat v5 package in R version 4.4.0. We excluded cells with fewer than 250 detected genes or larger than 20% mitochondrial genes. Seurat v5 was used to perform dimensionality reduction, clustering, and visualization. Recursive clustering analysis of subpopulations was conducted to improve the granularity of cell annotations and to obtain fibroblast cells. Cell-type annotation of fibroblasts was performed based on markers defined by Habermann. Visualization of the cells and clusters on a 2D map was performed with uniform manifold approximation and projection (UMAP). Dot plots and UMAP plots overlaid with gene expression levels were generated using Seurat.

### Statistical analysis

The qRT-PCR and metabolomic data were analyzed in Prism 10 (GraphPad Software, Inc). All data are shown as mean ± standard error of the mean. Significance was determined by one-way or two-way ANOVA using Tukey’s correction for multiple comparisons. * *P*<0.05, ** *P*<0.01, *** *P*<0.001.

## Supplementary material

Online supplementary figure 1

## Data Availability

Data presented in this manuscript will be made available upon request to the corresponding author. RNA-seq data generated from control and IPF HLFs are available in Gene Expression Omnibus (GEO) under accession number GSE273882. All code is available at https://github.com/MutluHamanakaLab. Metabolomics data are available via Figshare. https://dx.doi.org/10.6084/m9.figshare.28087574

## Abbreviations

AGC: automatic gain control
ARG1: Arginase 1
ARG2: Arginase 2
ASL: argininosuccinate lyase
ASS1: argininosuccinate synthase
GAPDH: glyceraldehyde-3-phosphate dehydrogenase
GCN2: general control nonderepressible 2
HLFs: human lung fibroblasts
HPLM: Human Plasma Like Medium
IPF: Idiopathic Pulmonary Fibrosis
MPA: mobile phase A
MPB: mobile phase B
NOS: nitric oxide synthases
OAT: ornithine amino transferase
P5C: pyrroline-5-carboxylate
SMAD: mothers against decapentaplegic homolog
TGF-β: transforming growth factor-β
TβR1: TGF-β receptor I
TβR2: TGF-β receptor II
UMAP: uniform manifold approximation and projection
VSMC: vascular smooth muscle cells
mTORC1: mechanistic target of rapamycin complex 1
α-SMA: α-smooth muscle actin
