# Supplementary material for: Arginine promotes the activation of human lung fibroblasts independent of its metabolism
Source: Biochem J. 2025 Jun 17;482(12):823–38. doi: 10.1042/BCJ20253033 (PMC12235046; doi:10.1042/BCJ20253033)
Supplement: Online supplementary figure 1 [file bcj-482-12-BCJ20253033-supp1.pdf]

Figure S1

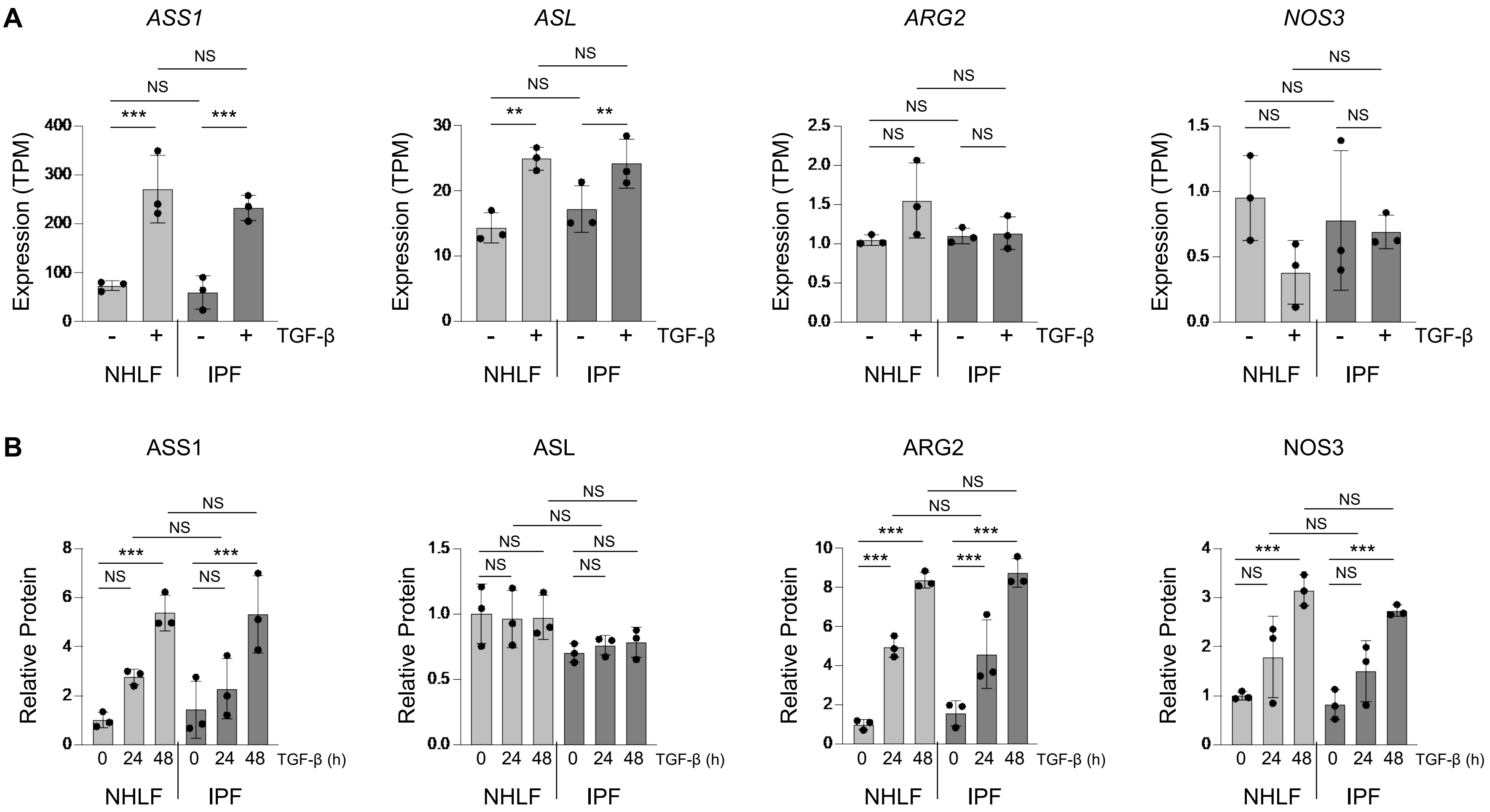

Figure S1. Analysis of arginine metabolic enzyme expression in HLFs after TGF-β. (A) Expression of *ASS1*, *ASL*, *ARG2*, and *NOS3* in 3 clones of normal HLFs and 3 clones of IPF HLFs in transcripts per million (TPM), derived from Kallisto quantification in Figure 1D. **(B)** Densitometric analysis of ASS1, ASL, ARG2, and NOS3 protein expression in 3 clones of normal HLFs and 3 clones of IPF HLFs from Figure 1E. Data are presented as mean±SD from 3 biologic replicates. * P<0.05, ** P<0.01, *** P<0.001 by two-way ANOVA using Tukey's post test.

Figure S2

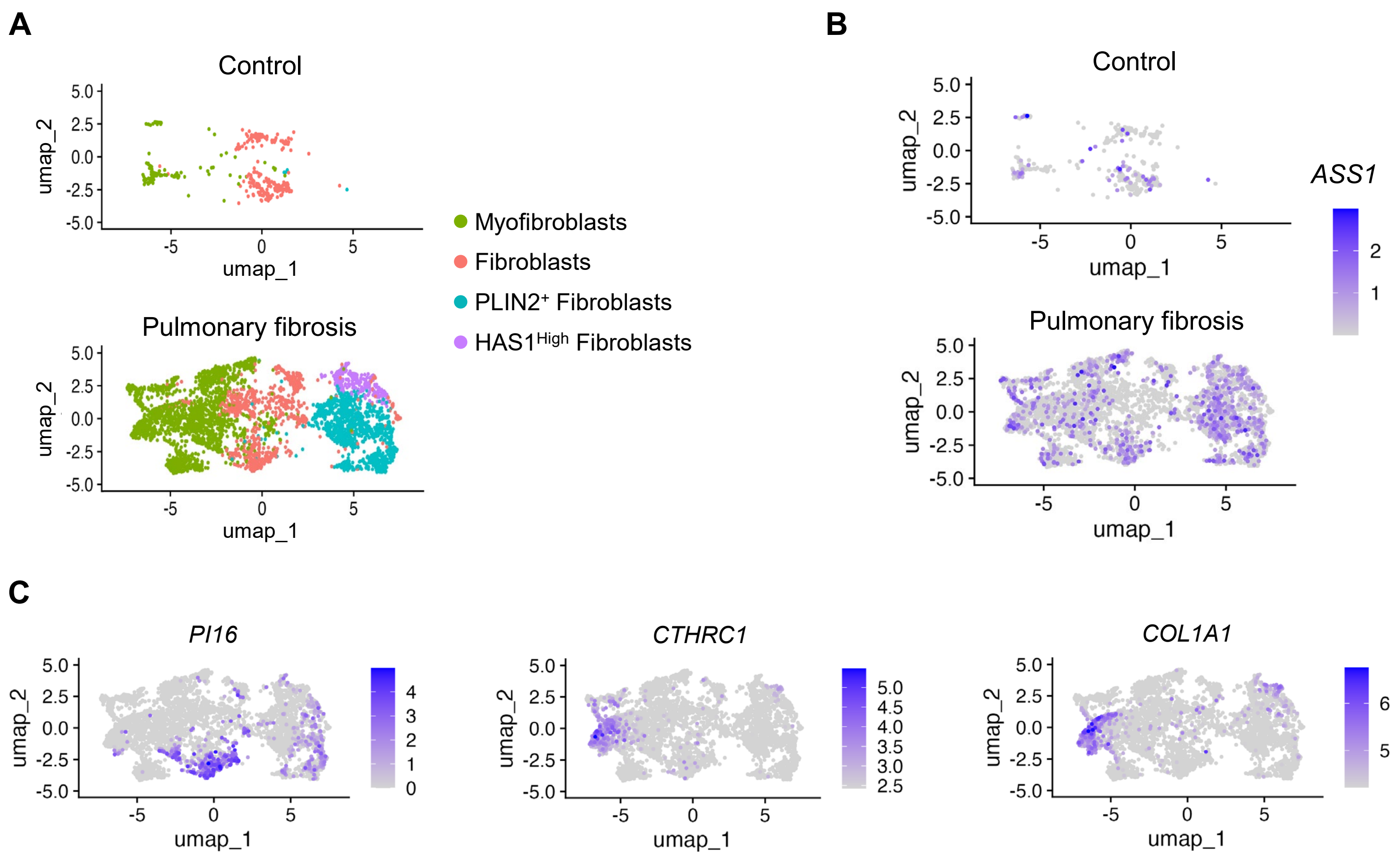

Figure S2. Fibroblast subpopulation gene expression. (A) Subclustering of myofibroblasts, fibroblasts, PLIN2$^+$ fibroblasts and HAS1^{High} fibroblasts from control donor lungs and from lungs from patients with pulmonary fibrosis segregated by disease status. **(B)** UMAP projection of the expression of *ASS1* mRNA in lung fibroblasts from control donor lungs and from lungs from patients with pulmonary fibrosis segregated by disease status. **(C)** Expression of *PI16* (adventitial fibroblast marker), *CTHRC1* (fibrotic fibroblast marker), and *COL1A1* in lung fibroblasts from control donor lungs and from lungs from patients with pulmonary fibrosis.

Figure S3

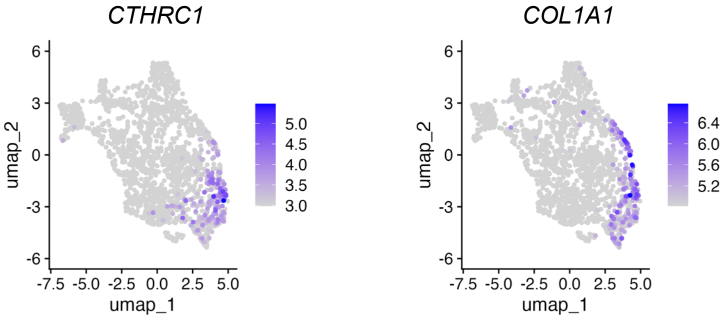

Figure S3. Expression of the fibrotic fibroblast markers. Expression of the fibrotic fibroblast markers, *CTHRC1* and *COL1A1* in subclustered alveolar and myofibroblast populations from Figure S2.

Figure S4

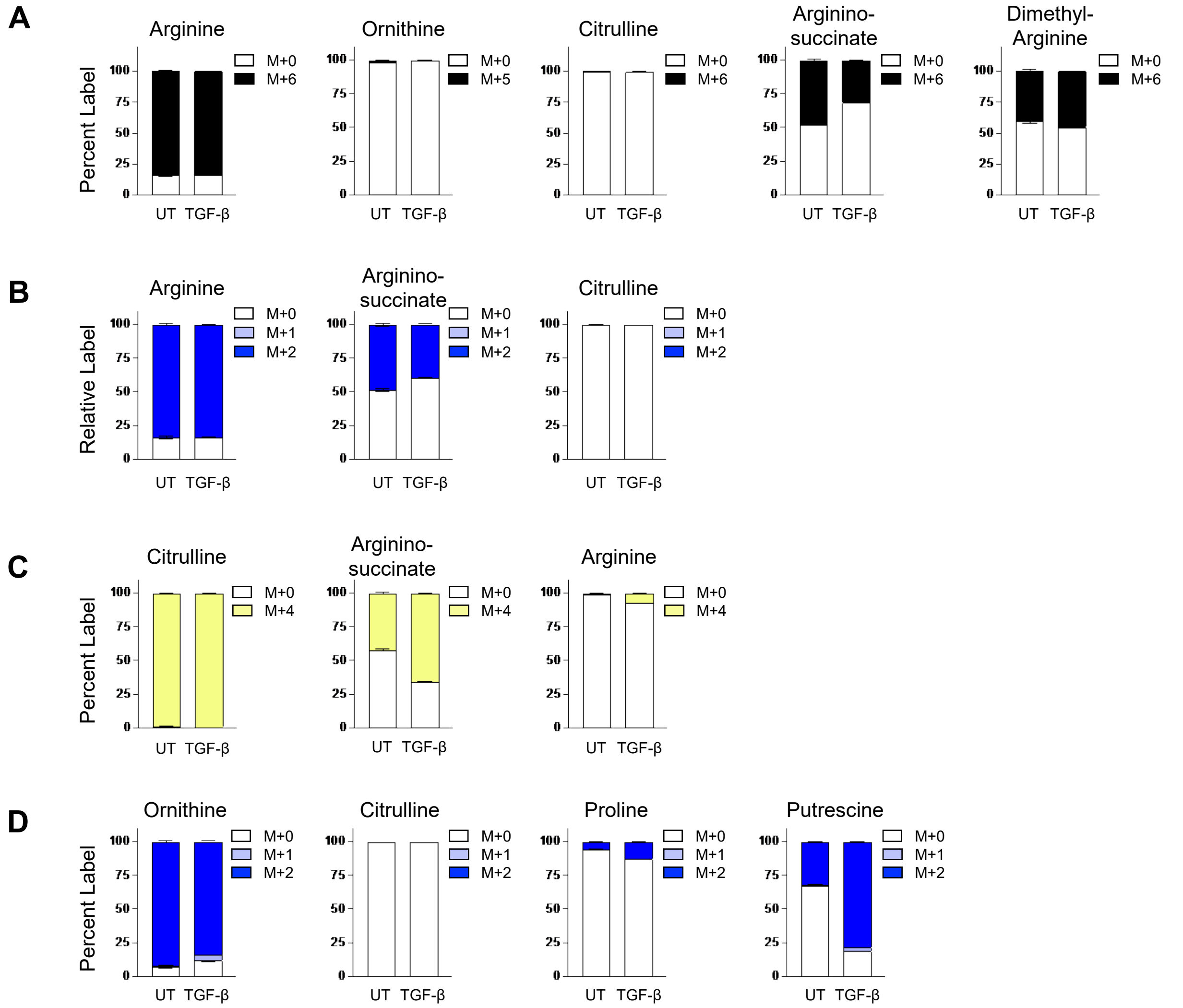

Figure S4. Percent labeling of arginine metabolism in human lung fibroblasts. (A) Analysis of cellular arginine, ornithine, citrulline, argininosuccinate, and dimethylarginine in HLFs after labeling with $^{13}C_6$ arginine HPLM in the presence or absence of TGF-β. **(B)** Analysis of cellular arginine, argininosuccinate, and citrulline in HLFs after labeling with guanido-$^{15}N_2$ arginine HPLM in the presence or absence of TGF-β. **(C)** Analysis of cellular citrulline, argininosuccinate, and arginine in HLFs after labeling with 4,4,5,5-D_4 citrulline HPLM in the presence or absence of TGF-β. **(D)** Analysis of cellular ornithine, citrulline, proline, and putrescine in HLFs after labeling with $^{15}N_2$ ornithine HPLM in the presence or absence of TGF-β. Data are presented as mean ± SEM from 4 biologic replicates.

Figure S5

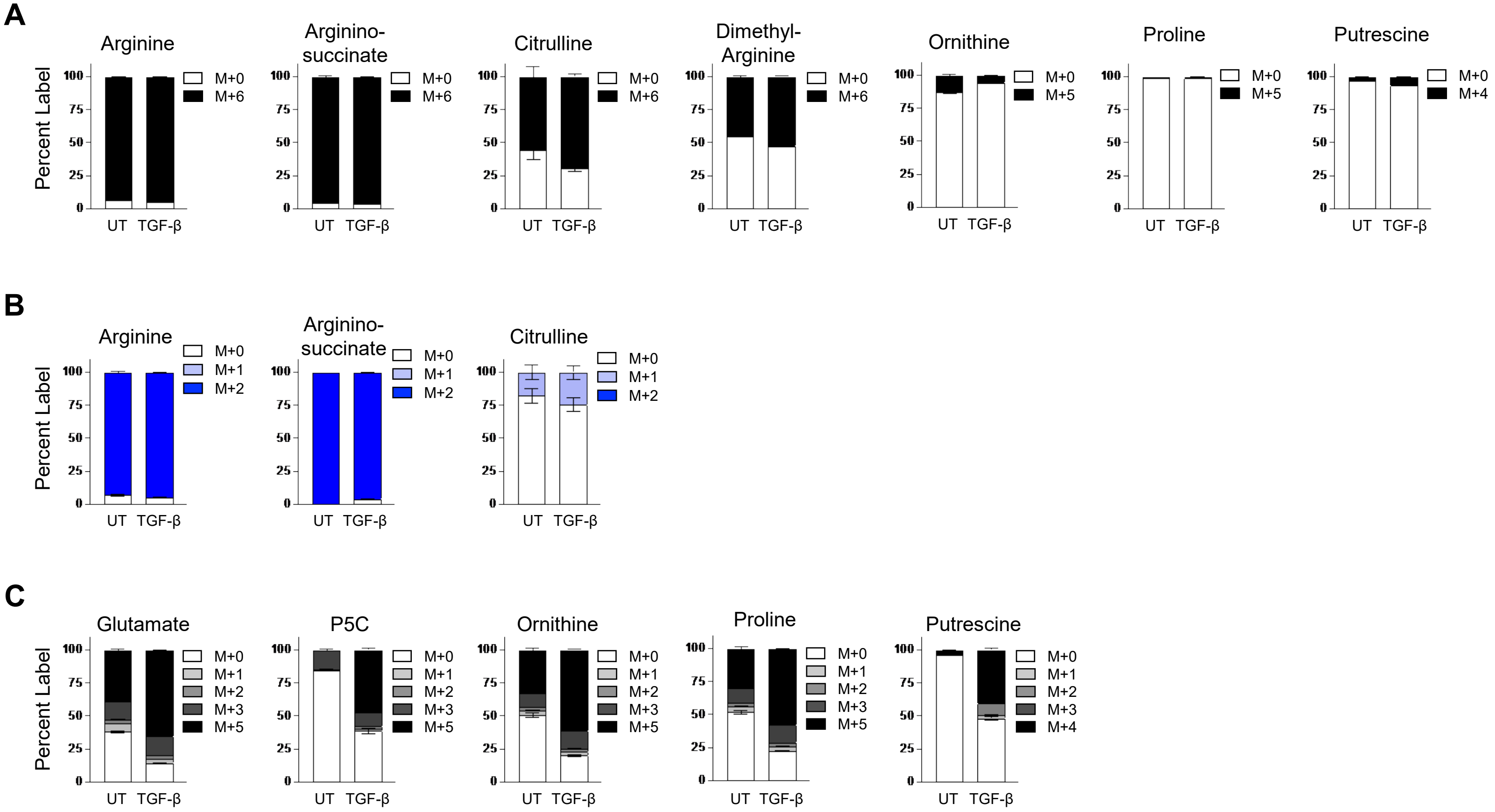

Figure S5. Percent labeling of arginine metabolism in human lung fibroblasts cultured in DMEM. (A) Analysis of cellular arginine, argininosuccinate, citrulline, dimethylarginine, ornithine, proline, and putrescine in HLFs after labeling with $^{13}C_6$ arginine DMEM in the presence or absence of TGF-β. **(B)** Analysis of cellular arginine, argininosuccinate, and citrulline in HLFs after labeling with guanido-^{15}N2 arginine DMEM in the presence or absence of TGF-β. **(C)** Analysis of cellular glutamate, pyrroline-5-carboxylate, ornithine, and putrescine in HLFs after labeling with $^{13}C_5$ glutamine DMEM in the presence or absence of TGF-β. Data are presented as mean±SD from 4 biologic replicates.

Figure S6

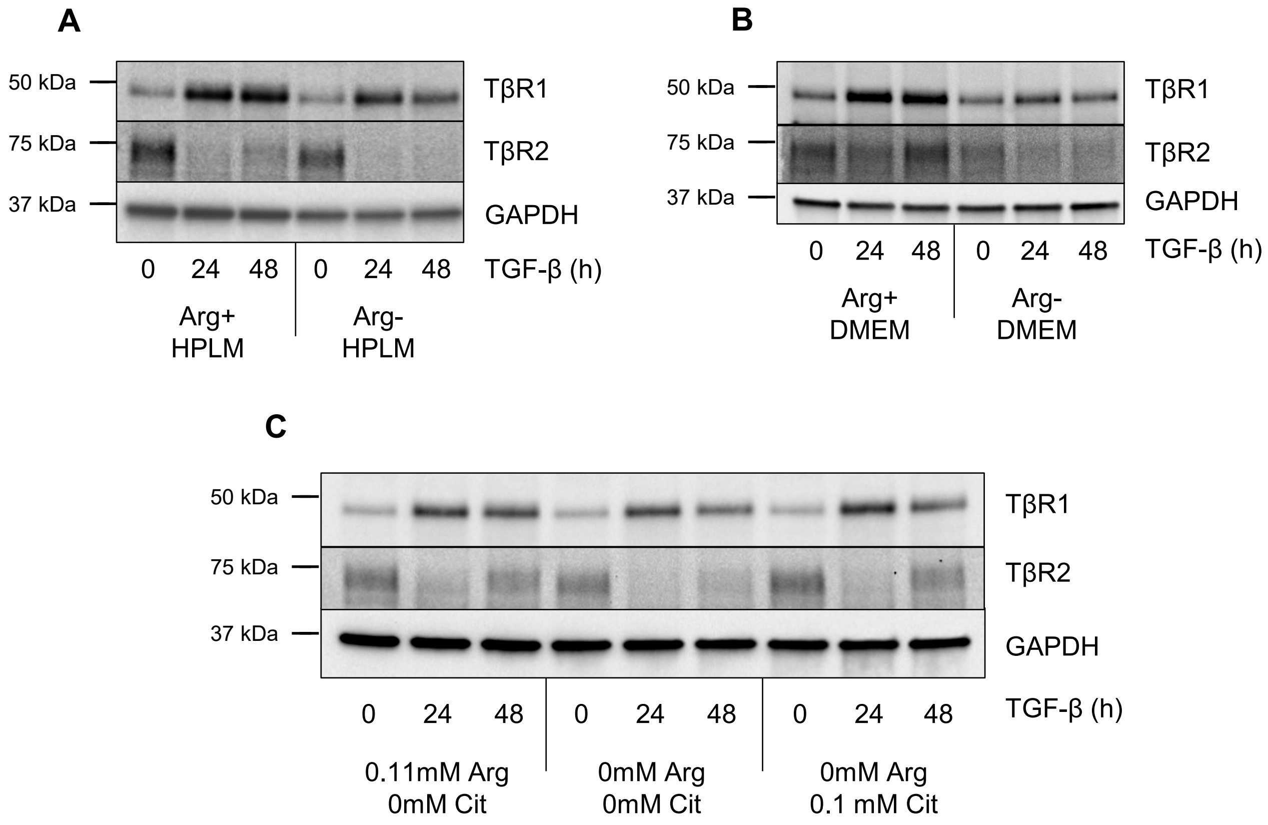

Figure S6. Regulation of TGF-β receptor expression by arginine in HLFs. (A-B) Western blot analysis of TGF-β receptor 1 (TβR1) and 2 (TβR2) in HLFs cultured in either (A) HPLM or (B) DMEM either containing or lacking arginine. Cells were treated with TGF-β for the indicated intervals. **(C)** Western blot analysis of TGF-β receptor 1 and 2 in HLFs cultured in HPLM containing 0.11mM Arg and 0mM Cit, 0mM Arg and 0mM Cit, or 0mM Arg and 0.1mM Cit.

Figure S7

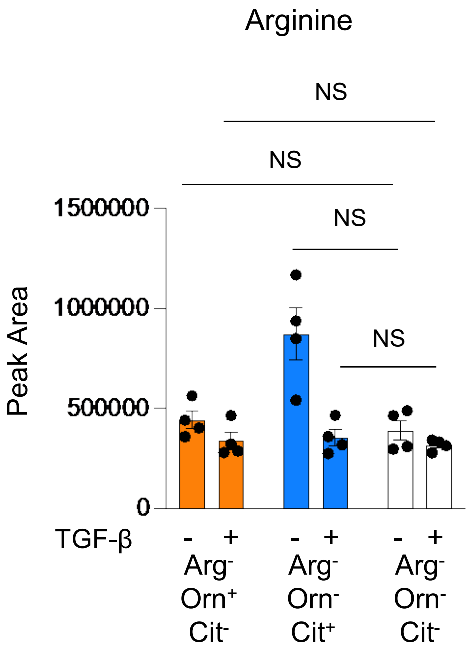

Figure S7. Extracellular citrulline contributes to intracellular arginine levels in the absence of extracellular arginine. Intracellular arginine levels in in HLFs cultured in arginine-free HPLM containing either ornithine (0.07mM), citrulline (0.04mM), or neither amino acid. Cells were treated with TGF-β or left untreated for 48 hours. Data are presented as mean±SEM from 4 biologic replicates. NS = not significant by two-way ANOVA using Tukey's post test.
